# Enhanced Rice Blast Resistance by CRISPR/Cas9-Targeted Mutagenesis of the ERF Transcription Factor Gene *OsERF922*

**DOI:** 10.1371/journal.pone.0154027

**Published:** 2016-04-26

**Authors:** Fujun Wang, Chunlian Wang, Piqing Liu, Cailin Lei, Wei Hao, Ying Gao, Yao-Guang Liu, Kaijun Zhao

**Affiliations:** 1 College of Agriculture, Guangxi University, Nanning, China; 2 National Key Facility for Crop Gene Resources and Genetic Improvement (NFCRI), Institute of Crop Science, Chinese Academy of Agriculture Sciences (CAAS), Beijing, China; 3 State Key Laboratory for Conservation and Utilization of Subtropical Agro-Bioresources, College of Life Sciences, South China Agricultural University, Guangzhou, China; University of Nebraska-Lincoln, UNITED STATES

## Abstract

Rice blast is one of the most destructive diseases affecting rice worldwide. The adoption of host resistance has proven to be the most economical and effective approach to control rice blast. In recent years, sequence-specific nucleases (SSNs) have been demonstrated to be powerful tools for the improvement of crops via gene-specific genome editing, and CRISPR/Cas9 is thought to be the most effective SSN. Here, we report the improvement of rice blast resistance by engineering a CRISPR/Cas9 SSN (C-ERF922) targeting the *OsERF922* gene in rice. Twenty-one C-ERF922-induced mutant plants (42.0%) were identified from 50 T_0_ transgenic plants. Sanger sequencing revealed that these plants harbored various insertion or deletion (InDel) mutations at the target site. We showed that all of the C-ERF922-induced allele mutations were transmitted to subsequent generations. Mutant plants harboring the desired gene modification but not containing the transferred DNA were obtained by segregation in the T_1_ and T_2_ generations. Six T_2_ homozygous mutant lines were further examined for a blast resistance phenotype and agronomic traits, such as plant height, flag leaf length and width, number of productive panicles, panicle length, number of grains per panicle, seed setting percentage and thousand seed weight. The results revealed that the number of blast lesions formed following pathogen infection was significantly decreased in all 6 mutant lines compared with wild-type plants at both the seedling and tillering stages. Furthermore, there were no significant differences between any of the 6 T_2_ mutant lines and the wild-type plants with regard to the agronomic traits tested. We also simultaneously targeted multiple sites within *OsERF922* by using Cas9/Multi-target-sgRNAs (C-ERF922S1S2 and C-ERF922S1S2S3) to obtain plants harboring mutations at two or three sites. Our results indicate that gene modification via CRISPR/Cas9 is a useful approach for enhancing blast resistance in rice.

## Introduction

Rice (*Oryza sativa* L.) is one of the most important food crops in the world, feeding nearly 50% of the world’s population. Rice blast, caused by the filamentous ascomycete fungus *Magnaporthe oryzae*, is one of the most destructive diseases affecting rice in all rice-growing countries and often causes serious damage to global rice production [1, 2]. Enhancing the resistance of rice to *M*. *oryzae* has been shown to be the most economical and effective approach for controlling rice blast [3, 4].

Over the course of evolution, plants have evolved sophisticated mechanisms to resist pathogen infection. In plant cells, surface-localized pattern recognition receptors (PRRs) rapidly perceive pathogen-associated molecular patterns (PAMPs) [5] and activate a battery of defense mechanisms [6]. PAMP-triggered immunity (PTI) is considered a conserved and ancient form of plant immunity that acts as the first line of inducible defense to various pathogens [6, 7]. The plant hormones abscisic acid, salicylic acid, jasmonic acid and ethylene play important roles in this defense response [8–10].

Plant ethylene responsive factors (ERF), a subfamily of the APETELA2/ethylene response factor (AP2/ERF) transcription factor superfamily in plants, are involved in the modulation of multiple stress tolerance and have been implicated in multiple responses to abiotic and biotic stresses [11–13]. For example, expression of the rice ERF genes *OsBIERF1*, *OsBIERF3* and *OsBIERF4* are not only induced by *M*. *oryzae* infection, but also up-regulated by salt, cold, drought and wounding [14]. Likewise, the overexpression of *BrERF11* enhanced disease resistance to *Ralstonia solanacearum* in tobacco [15]. However, overexpression of *PpERF3b* in tobacco contributes to susceptibility to disease caused by *Pseudomonas syringae* pv. *Tabaci* [16]. This is in line with the finding that silencing *StERF3* in potato produced enhanced foliage resistance to *Phytophthora infestans* [17]. Similarly, knockdown of expression of the rice ERF gene *OsERF922* by RNA interference (RNAi) enhanced rice resistance to *M*. *oryzae*, indicating that OsERF922 acts as a negative regulator of blast resistance in rice [18].

In conventional rice breeding, it takes approximately a decade to pyramid multiple blast resistance genes into a rice variety via crossing and backcrossing, while the high pathogenic variability in *M*. *oryzae* often leads to the rapid break down of resistant cultivars [19]. Thus, enhancement of PTI for the development of broad-spectrum resistance has been suggested as an effective approach for breeding varieties resistant to rice blast [20–22]. Breeding strategy using RNAi-based down-regulation of rice transcription factor expression has been demonstrated to be an alternative approach for enhancing rice resistance to blast [18, 23–25]. However, expression of RNAi transgenes varies in different transgenic lines; a large number of transgenic plants are required to identify candidates in which the transgene is highly expressed over multiple generations. In addition, rice plants derived by RNAi methods are usually regarded as transgenic and are subjected to rigorous regulatory processes [26].

In recent years, sequence-specific nucleases (SSNs), including zinc finger nucleases (ZFNs), transcription activator-like effector nucleases (TALENs) and clustered regularly interspaced short palindromic repeats (CRISPR)/CRISPR-associated (Cas) 9 (CRISPR/Cas9) have been demonstrated to be useful tools for plant genome editing [27, 28]. In particular, CRISPR/Cas9 has been demonstrated to be the most effective SSN thus far and has been used for genome editing in major crops such as rice [29–41], maize [42–45], wheat [39, 46, 47], sorghum [30], tomato [48, 49], soybean [50–52] and potato [53].

The gene-specific DNA double-strand breaks (DSBs) caused by the SSNs are repaired primarily by the high-fidelity homologous recombination (HR) or error-prone non-homologous end joining (NHEJ) pathways [54]. NHEJ often introduces small insertion or deletion (InDel) mutations at the cut site that lead to the loss of gene function. Compared with RNAi technology, SSN-based genome editing can achieve complete knockout without incorporating exogenous DNA. To date, successful examples of ZFN- and TALEN-based improvements of agronomically important traits in major crops have been reported [47, 55–60]. Here, we report the improvement of rice blast resistance via CRISPR/Cas9-targeted knockout of the ERF transcription factor gene *OsERF922* in Kuiku131, a *japonica* rice variety widely cultivated in northern China.

## Materials and Methods

### Plant growth

The *japonica* rice variety Kuiku131 and all transgenic plants were grown in a net house and greenhouse at 28–35°C in Beijing, or fields in the experimental station under normal growth conditions in Sanya. The experimental station is specialized for genetically modified crops planting permitted by Chinese Ministry of Agriculture. For blast inoculation at the seedling stage, rice seeds were grown in 60 × 30 × 5 cm plastic seedling-nursing trays supplemented with a mineral nutrient solution in a greenhouse maintained at approximately 28–35°C under natural sunlight [61]. Briefly, moistened seeds of wild-type rice and rice mutants (T_2_ progeny) were sown in rows (15 seed per row) in triplicate trays.

### Vector construction

**Fig 1:**
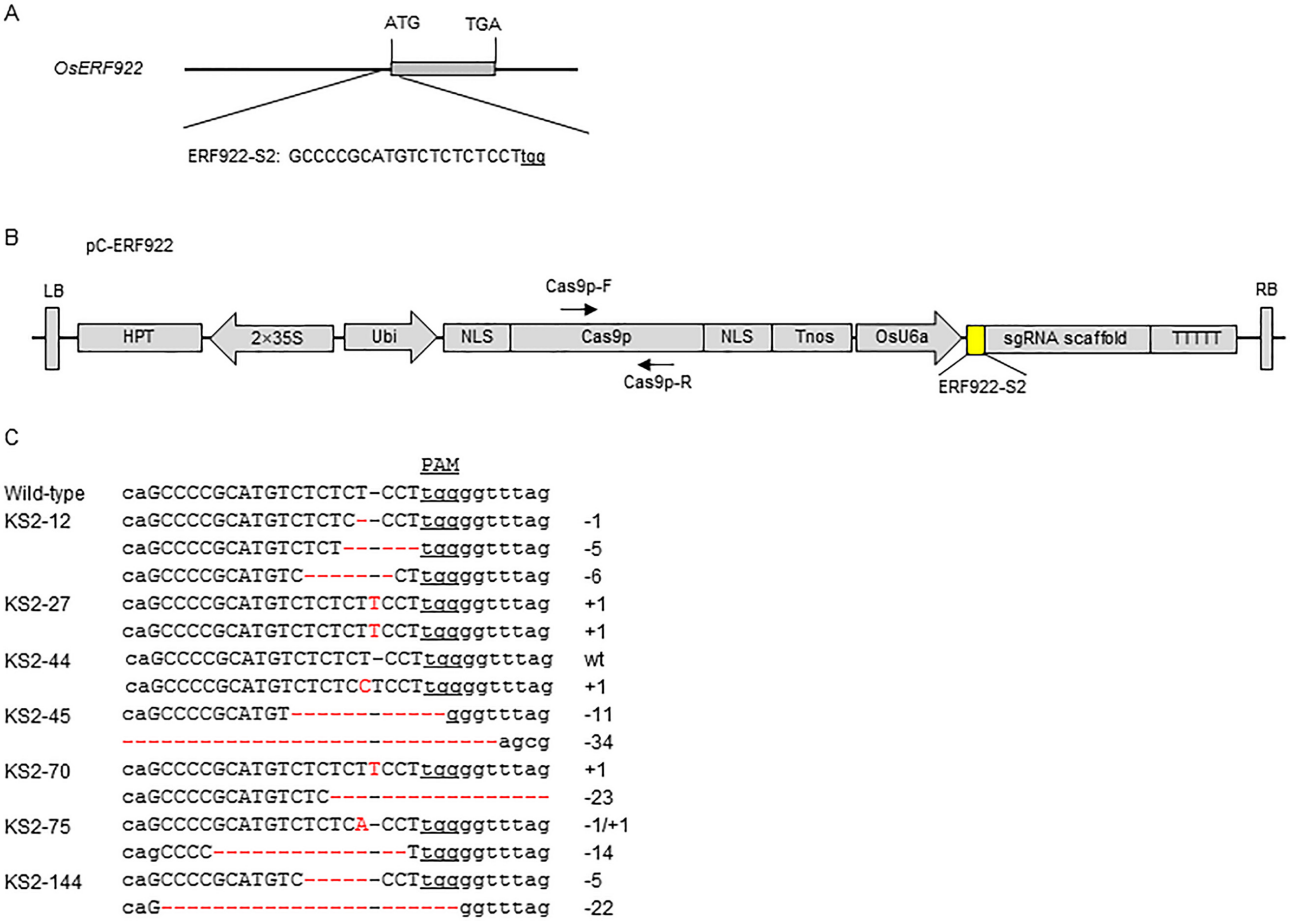
CRISPR/Cas9-induced *OsERF922* gene modification in rice. (A) Schematic of *OsERF922* gene structure and the C-ERF922 target site (ERF922-S2). *OsERF922* contains a single exon indicated by gray rectangles. The translation initiation codon (ATG) and termination codon (TGA) are shown. The target site nucleotides are shown in capital letters, and the protospacer adjacent motif (PAM) site is underlined. (B) Schematic diagram of the pC-ERF922 construct for expressing the CRISPR/Cas9 protein C-ERF922. The positions and orientations of the primers Cas9p-F and Cas9p-R, which were used to screen Cas9-free mutants, are indicated by small arrows. The expression of *Cas9* is driven by the maize ubiquitin promoter (Ubi); the expression of the sgRNA scaffold is driven by the rice U6a small nuclear RNA promoter (OsU6a); the expression of *hygromycin* (HPT) is driven by 2 CaMV35S promoters (2 × 35S). NLS: nuclear localization signal; Tnos: gene terminator; LB and RB: left border and right border, respectively. (C) Nucleotide sequences at the target site in the 7 T_0_ mutant rice plants. The recovered mutated alleles are shown below the wild-type sequence. The target site nucleotides are indicated as black capital letters and black dashes. The PAM site is underlined. The red dashes indicate the deleted nucleotides. The red capital letters indicate the inserted nucleotides. The numbers on the right indicate the type of mutation and the number of nucleotides involved. “−” and “+” indicate the deletion and insertion of the indicated number of nucleotides, respectively; “−/+” indicates the simultaneous deletion and insertion of the indicated number of nucleotide.

The C-ERF922-expressing vector (pC-ERF922) was constructed according to a method described by Ma et al. [38]. Briefly, the target site (Fig 1A) sequence-containing primers E922-FS2/E922-RS2 (S1 Table) were synthesized by Sangon Biotech (http://www.sangon.com/) and combined by annealing. Then, the target site sequence-containing chimeric primers were cloned into the sgRNA expression cassette pYLsgRNA-U6a at a *Bsa*I site [38]. The integrated sgRNA expression cassette was then amplified by nested PCR [38] using the primers U-F/E922-RS2 and E922-FS2/gR-R (for the first round) and the site-specific primers Pps-GGL and Pgs-GGR (for the second round) (S1 Table). Subsequently, the amplicons containing ERF922-S2-sgRNA with different *Bsa*I-cutting sites were cloned into the CRISPR/Cas9 Multi-targeting vector pYLCRISPR/Cas9Pubi-H at a *Bsa*I site [38]; the resultant construct pC-ERF922 contained a Cas9p expression cassette (Pubi::NLS::Cas9p::NLS::Tnos) and a hygromycin resistance cassette (2×P35S::HPT::T35STnos) (Fig 1B).

The C-ERF922S1S2- and C-ERF922S1S2S3-expressing vectors (pC-ERF922S1S2 and pC-ERF922S1S2S3) were constructed according to the method described by Ma et al. [38]. Briefly, the target site-containing sequence primers E922-FS1/E922-RS1, E922-FS2/E922-RS2 and E922-FS3/E922-RS3 (S1 Table, Fig 1A and S1 Fig) were combined by annealing, and then the target site sequence-containing chimeric primers were cloned into the sgRNA expression cassettes pYLsgRNA-U3, pYLsgRNA-U6a and pYLsgRNA-U6b at a *Bsa*I site [38], respectively. The integrated sgRNA expression cassettes were then amplified by nested PCR [38] using U-F/Reverse adapter primers (E922-RS1, E922-RS2 and E922-RS3) and Forward adapter primers (E922-FS1, E922-FS2 and E922-FS3)/gR-R for the first round, and the corresponding site-specific primers Pps-GGL/Pgs-GG2, Pps-GG2/Pgs-GGR and Pps-GGL/Pgs-GG2, Pps-GG2/Pgs-GG3, Pps-GG3/Pgs-GGR for the second round (S1 Table). Subsequently, the two-target-sgRNAs (ERF922-S1-sgRNA and ERF922-S2-sgRNA) and three-target-sgRNAs (ERF922-S1-sgRNA, ERF922-S2-sgRNA and ERF922-S3-sgRNA) expression cassettes were ligated into the pYLCRISPR/Cas9Pubi-H vector [38], resulting in the C-ERF922S1S2- and C-ERF922S1S2S3-expressing constructs pC-ERF922S1S2 and and C-ERF922S1S2S3-expressing constructs pC-ERF922S1S2 and pC-ERF922S1S2S3 (S2 Fig).

### Rice transformation

The Cas9/sgRNA-expressing binary vectors (pC-ERF922, pC-ERF922S1S2 and pC-ERF922S1S2S3) were transformed into an *Agrobacterium tumefaciens* strain EHA105 by electroporation. *Agrobacterium*-mediated transformation of the embryogenic calli derived from the *japonica* rice variety Kuiku131 was performed according to Hiei et al. [62]. Briefly, hygromycin-containing medium was used to select hygromycin-resistant calli, and then the hygromycin-resistant calli were transferred onto regeneration medium for the regeneration of transgenic plants. After 2–3 months of cultivation, transgenic seedlings were transferred to a field during the rice growing season.

### Protoplast assay

Kuiku131 seeds were sterilized with 0.2% potassium permanganate solution for 24 h and then imbibed in water at 37°C for 24 h before germination. Seedlings were grown in cylindrical glass bottles lined with wet toilet paper under a regime of 12 h light (150–200 μmol m^-2^ s^-1^)/12 h dark at 26°C in an incubator for 10–14 days before protoplast isolation. The protoplast isolation and transformation were performed following protocols published by Zhang et al. [63] and Shan et al. [64], respectively.

Healthy and fresh rice stems and sheaths from 50 rice plants were used. A bundle of rice plants were cut together into fine strips approximately 0.5 mm in length using sharp razors. The fine strips were immediately transferred into an enzyme solution (1.5% Cellulase RS, 0.75% Macerozyme R-10, 0.6 M mannitol, 10 mM MES at pH 5.7, 10 mM CaCl_2_ and 0.1% BSA). After 5 h digestion with gentle shaking (60–80 rpm) in the dark, an equal volume of W5 solution (154 mM NaCl, 125 mM CaCl_2_, 5 mM KCl and 2 mM MES at pH 5.7) was added, followed by light shaking for 10 sec. The protoplasts were released by filtering through 40 μm nylon meshes into round bottom tubes and were washed 2–3 times using W5 solution. The pellets were collected by centrifugation at 250 g for 3 min. After washing, the pellets were resuspended in MMG solution (0.4 M mannitol, 15 mM MgCl_2_ and 4 mM MES at pH 5.7) at a concentration of 2 × 10^6^ cells mL^-1^, as calculated using a hematocytometer.

Protoplast transformation was carried out in a poly-ethylene glycol (PEG) solution [40% (W/V) PEG 4000, 0.2 M mannitol and 0.1 M CaCl_2_]. For one sample, 20 μg of plasmid DNA was mixed with 200 μL protoplasts (approximately 4 × 10^5^ cells) and 220 μl freshly prepared PEG solution, and the mixture was incubated at room temperature for 20 min in the dark. After incubation, 880 μL of W5 solution was added slowly, and the protoplast cells were harvested by centrifugation at 250 g for 3 min. The protoplast cells were resuspended gently in 2 mL WI solution (0.5 M mannitol, 20 mM KCl and 4 mM MES at pH 5.7) and cultured in 6-well plates in darkness at room temperature for 48 h.

The genomic DNA was extracted from protoplast cells transformed with pC-ERF922 plasmid by the SDS method [65]. The protoplast genomic DNA was subjected to PCR with the gene-specific primer pair E922-KF/E922-KR (S1 Table) to amplify DNA fragments across the target site. Then, the PCR amplicons were cloned into the pEASY-Blunt vector (TransGen Biotech, Beijing, China), and a total of 48 randomly selected colonies were further characterized by Sanger DNA sequencing.

### Identification of mutant transgenic plants

Genomic DNA was extracted from individual transgenic plants using SDS extraction according to Dellaporta et al. [65]. All transgenic hygromycin-resistant T_0_ plants were characterized by PCR using the *Cas9*-specific primers Cas9p-F/Cas9p-R (Fig 1B and S1 Table). Subsequently, all PCR-positive plants were subjected to PCR using the gene-specific primer pairs E922-KF/E922-KR and E922P-2F/E922-KR2 (S1 Table) to amplify DNA fragments across the target sites (ERF922-S2, ERF922-S1, ERF922-S2 and ERF922-S3, respectively); the PCR amplicons were then directly sequenced. The sequencing chromatograms with superimposed peaks of bi-allelic and heterozygous mutations were decoded by the Degenerate Sequence Decoding (DSD) method [66]-based web tool DSDecode (http://dsdecode.scgene.com/) [67].

### Pathogen inoculation

To evaluate the resistance to *M*. *oryzae* at the seedling stage, the inoculation of rice blast fungus *M*. *oryzae* was performed according to a method described by Wang et al. [20]. Briefly, 3–4 week-old wild-type and homozygous mutant plants were inoculated by spraying with conidial suspensions (2 × 10^5^ conidia mL^-1^, 0.02% Tween 20) of *M*. *oryzae* isolate 06-47-6. The inoculated plants were grown in an ENCONAIR phytotron at 26°C (95% humidity) in the dark for 24 h, then were grown under conditions of a 16 h/8 h light/dark cycle with 95% humidity. Disease severity was evaluated according to the method described by Fukuoka et al. [68] at 7 d post inoculation (dpi). The area of the lesions was determined for the third leaves of 10 plants of each line. The experiments were repeated three times.

To confirm the presence of disease, all seedlings of wild-type and homozygous mutant lines were transplanted to the field and injection-inoculated at the tillering stage according to a method described by Ma et al. [69]. Briefly, when rice plants grow to 5–6 tillers, conidial suspensions (2 × 10^4^ conidia mL^-1^) of *M*. *oryzae* were injected into the rice stem with a syringe until the suspension emerged from the heart-leaf. The injection sites on the stems were approximately 10 cm from the top of the tillers. Five tillers were injected for each plant. Disease severity was evaluated according to method described by Kobayashi et al. [70] at 7 dpi. The length of the lesions was assessed on the inoculated leaves of five tillers for each line. The experiments were repeated three times.

### Agronomic trait characterization

Wild-type and homozygous T_2_ mutant lines were grown in the field under normal growth conditions in Beijing. Agronomic traits were characterized by measuring plant height, flag leaf length and width, the number of productive panicles, panicle length, the number of grains per panicle, the seed setting rate, and thousand seed weight after the rice had reached maturity. Five plants were investigated for each line.

## Results

### CRISPR/Cas9 design and the assessment of gene-editing activity

To design a CRISPR/Cas9 (C-ERF922) targeting the *OsERF922* gene in rice, a 20-bp nucleotide sequence containing the initiation codon of the open reading frame of *OsERF922* was chosen as the target site (ERF922-S2) (Fig 1A). The predicted Cas9 cleavage site in the coding region of the gene was seven base-pairs downstream from the ATG initiation codon. The binary plasmid pC-ERF922 (Fig 1B) was then constructed based on the CRISPR/Cas9 vector described by Ma et al. [38]. To test the gene-editing efficacy of C-ERF922, rice protoplasts were transformed with pC-ERF922, and genomic DNA was extracted to amplify the DNA fragment containing the target site. PCR amplicons generated with the primers E922-KF and E922-KR (S1 Table) were cloned into the pEASY-Blunt vector to isolate the colonies for sequencing. Three mutants (6.3%, S3 Fig) were recovered from 48 randomly selected colonies. Sequencing revealed that the mutation in colony C1 was a single nucleotide substitution; in colony C2 was a 5-bp deletion; and in colony C3 was a 30-bp insertion (S3 Fig). These observations showed that the C-ERF922-expression construct in pC-ERF922 exhibits gene-editing activity in rice protoplasts and can be used for creating mutant rice plants.

### Recovery of rice plants with mutations in *OsERF922*

The pC-ERF922 construct was used to transform the rice variety Kuiku131 by *Agrobacterium*-mediated transformation, with the goal of enhancing its blast resistance by gene-specific editing. We obtained 50 positive transgenic (T_0_) plants and analyzed the target site in 21 of the plants (S4 Fig). Direct Sanger-sequencing of the target-containing amplicons followed by decoding via the DSD method [66, 67] showed that among the 21 plants, there were 16 bi-allelic mutations, 3 homozygous mutations, 1 heterozygous mutation, and 1 chimeric mutation (Table 1). Based on allele mutation types, more than half (64.3%, 27/42) of the mutations were nucleotide deletions, 23.8% (10/42) of the mutations were nucleotide insertions, and 11.9% (5/42) of the mutations were simultaneous nucleotide deletions and insertions (Table 1). As for the deletion mutations, the majority (63.0%, 17/27) were short (≤ 10 bp) deletions and the other 37.0% (10/27) were longer deletions ranging from 11 bp to 34 bp (S4 Fig); as for the insertion mutations, 90.0% (9/10) were 1 bp insertions (S4 Fig).

**Table 1:** Ratios of mutant genotype and mutation type at the target site (ERF922-S2) in T_0_ mutant plants.

Mutant genotype ratios (%) ^a^				Mutation type ratios (%) ^b^		
Chimera*	Bi-allele	Homozygote	Heterozygote	Deletion	Insertion	Deletion and insertion
4.8(1/21)	76.1 (16/21)	14.3(3/21)	4.8 (1/21)	64.3 (27/42)	23.8 (10/42)	11.9 (5/42)

^a^ Based on the number of each mutant genotype out of the total number of all mutant genotypes at the target site.

^b^ Based on the number of each allele mutation type out of the total number of all allele mutation types at the target site.

* Refers to a plant with at least three distinct alleles detected at the target site.

### Transmission of C-ERF922-induced mutations from the T_0_ to the T_1_ and T_2_ generations

To determine whether and how the C-ERF922-induced mutations were transmitted to the next generation, 4 bi-allelic (KS2-45, 70, 75, 144), 1 chimeric (KS2-12), 1 homozygous (KS2-27) and 1 heterozygous (KS2-44) T_0_ mutant plants (Fig 1C) were self-pollinated, and their progenies were genotyped at the target site. A total of 120 T_1_ plants derived from the T_0_ mutant plants were genotyped by PCR and DNA sequencing (Table 2). We found that all allelic mutations in the T_0_ mutant plants were transmitted to the T_1_ generation with a transmission rate of 100% (Table 2). In theory, allelic mutations in the bi-allelic T_0_ mutant plants should segregate to T_1_ plants following Mendelian genetic law (1xx:2xy:1yy). As expected, homozygous genotypes were detected in all T_1_ populations derived from the T_0_ mutant plants, even when the T_1_ segregation pattern of progeny from the chimeric T_0_ mutant plant KS2-12 was more diverse and less predictable. For all bi-allelic T_0_ mutant plants with the exception of KS2-45, the segregation ratio of [homozygote (xx): bi-allele mutants (xy): homozygote (yy)] in the T_1_ populations fit a 1:2:1 ratio shown to be statistically reliable in a chi-square test of the T_1_ plants (Table 2). For example, the bi-allelic T_0_ mutant plant KS2-70 harbors two mutations [a 23-bp deletion (-23) and a 1-bp insertion (+1)]; its T_1_ progenies segregated in a ratio of [11(-23): 20(-23, +1): 10(+1)], matching the (1xx: 2xy: 1yy) ratio well (Table 2).

**Table 2:** Segregation and types of C-ERF922-induced mutations in the target gene and their transmission to subsequent generations.

			Mutation transmission in the T_1_ or T_2_ generations						
Mutant plants ^a^	Genotype	Mutation type ^b^	No. of plants tested	Wt	Bi-allele	Homozygote	Heterozygote	Transmission ratio (%) ^c^	No. of T-DNA-free plants ^d^
**T**_**0**_ **generation**									
**KS2-12**	Chimera*	-1, -5, -6	8	0	2(-1, -5), 2(-1, -6)	4(-1)	0	100.0	0
**KS2-27**	Homozygote	+1	6	0	0	6(+1)	0	100.0	1
**KS2-44**	Heterozygote	+1, wt	3	0	0	2(+1)	1(+1, wt)	100.0	0
**KS2-45**	Bi-allele	-11, -34	37	0	26(-11, -34)	3(-11), 8(-34)	0	100.0	3
**KS2-70**	Bi-allele	-23, +1	41	0	20(-23, +1)	11(-23), 10(+1)	0	100.0^e^	6
**KS2-75**	Bi-allele	-14, -1/+1	14	0	5(-14, -1/+1)	4(-14), 5(-1/+1)	0	100.0^e^	0
**KS2-144**	Bi-allele	-5, -22	11	0	5(-5, -22)	3(-5), 3(-22)	0	100.0^e^	0
**T**_**1**_ **generation**									
**KS2-27-1**	Homozygote	+1	30	0	0	30(+1)	0	100.0	7
**KS2-44-1**	Heterozygote	+1, wt	40	12	4(-1/+1, -1), 2(-1, +1)	14(+1)	8(+1, wt)	70.0	0
**KS2-45-1**	Bi-allele	-11, -34	57	0	28(-11, -34)	10(-11), 19(-34)	0	100.0^e^	9
**KS2-45-2**	Homozygote	-11	30	0	0	30(-11)	0	100.0	0
**KS2-45-6**	Homozygote^#^	-34	30	0	0	30(-34)	0	100.0	30

^a^ KS2-27-1 and KS2-44-1 were progenies of KS2-27 and KS2-44, respectively; KS2-45-1, KS2-45-2 and KS2-45-6 were progenies of KS2-45.

^b^ “−” indicates the deletion of the indicated number of nucleotides; “+” indicates the insertion of the indicated number of nucleotides; “−/+” indicates the simultaneous deletion and insertion of the indicated number of nucleotides at the same site.

^c^ Based on the number of plants carrying the observed mutation out of the total number of plants tested.

^d^ Mutant plants not containing DNA from the pC-ERF922 construct.

^e^ Segregation of the bi-allele lines conforms to a Mendelian 1: 2: 1 ratio according to the χ2 test (P > 0.5).

* Refers to a plant with at least three distinct alleles detected at the target site

^#^ T-DNA-free homozygote.

The transmission of mutations from T_1_ mutant plants to the T_2_ generation was further investigated using 3 homozygous (KS2-27-1, KS2-45-2 and KS2-45-6), 1 bi-allelic (KS2-45-1) and 1 heterozygous (KS2-44-1) T_1_ mutant plants (Table 2). Similarly, all allelic mutations in the T_1_ mutant plants were transmitted to the T_2_ generation, and the transmission rates ranged from 70% to 100%; the segregation pattern of T_2_ plants derived from the bi-allelic T_1_ mutant plant KS2-45-1 also followed Mendelian genetic law (Table 2). In addition, all T_1_ plants derived from the homozygous T_0_ mutant plant (KS2-27) and T_2_ plants derived from homozygous T_1_ mutant plants (KS2-27-1, KS2-45-2 and KS2-45-6) were homozygous for the same mutations (Table 2), indicating that the mutations in these homozygous mutant lines were stably transmitted to the next generation as expected. Interestingly, several new allelic mutations [(-1/+1, -1) and 2(-1, +1)] were detected among the T_2_ progeny of KS2-44-1 (Table 2 and S5 Fig), revealing that C-ERF922 activity was sustainable in the heterozygous T_1_ mutant plant. Together, these results clearly demonstrate that CRISPR/Cas9-induced gene mutations can be stably transmitted to subsequent generations.

### Selection of T-DNA-free mutant rice lines

**Fig 2:**
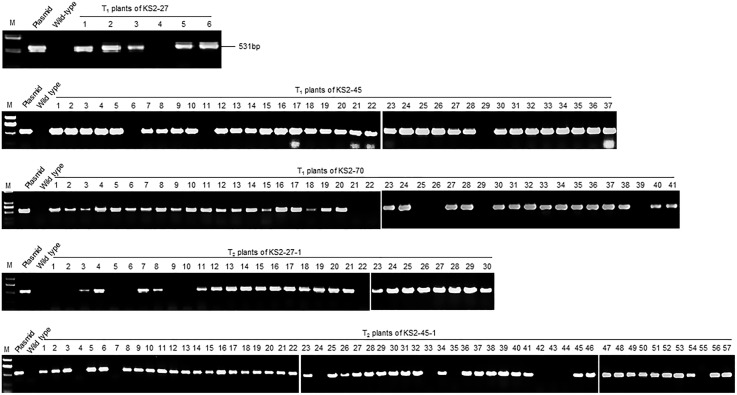
PCR-based identification of T-DNA-free rice mutant plants. PCR products amplified from the progenies of KS2-27, KS2-45, KS2-70, KS2-27-1 and KS2-45-1 genomic DNA using the primers Cas9p-F and Cas9p-R. The numbers above the gel image refer to individual offspring of KS2-27, KS2-45, KS2-70, KS2-27-1 and KS2-45-1, respectively. M: DNA molecular weight marker; Plasmid: pC-ERF922; Wild-type: genomic DNA from Kuiku131.

To investigate the possibility of obtaining rice lines harboring the desired modifications in *OsERF922* but without transferred DNA (T-DNA) of the construct pC-ERF922, we designed the *Cas9* gene-specific PCR primers Cas9p-F and Cas9p-R (Fig 1B and S1 Table) and performed PCR assays of the T_1_ and T_2_ plants. All 120 T_1_ plants were subjected to PCR assays, and 10 (8.3%) T_1_ plants failed to generate a *Cas9*-specific 531-bp amplicon from the transferred pC-ERF922 construct (Fig 2 and Table 2). Similarly, the PCR assay also failed to detect the pC-ERF922 construct in 16 out of 157 (10.2%) T_2_ plants derived from the 4 T_1_ mutant plants (KS2-27-1, KS2-44-1, KS2-45-1 and KS2-45-2) (Fig 2 and Table 2). Notably, all 30 T_2_ plants derived from the T_1_ mutant plant KS2-45-6 failed to generate the *Cas9*-specific amplicon (Table 2) because the KS2-45-6 plant was a Cas9-free homozygous mutant harboring the desired *OsERF922* modifications. These results indicate that T-DNA-free plants carrying the desired gene modifications can be acquired through genetic segregation.

### Resistance to *M*. *oryzae* was enhanced in C-ERF922-induced rice mutants

**Fig 3:**
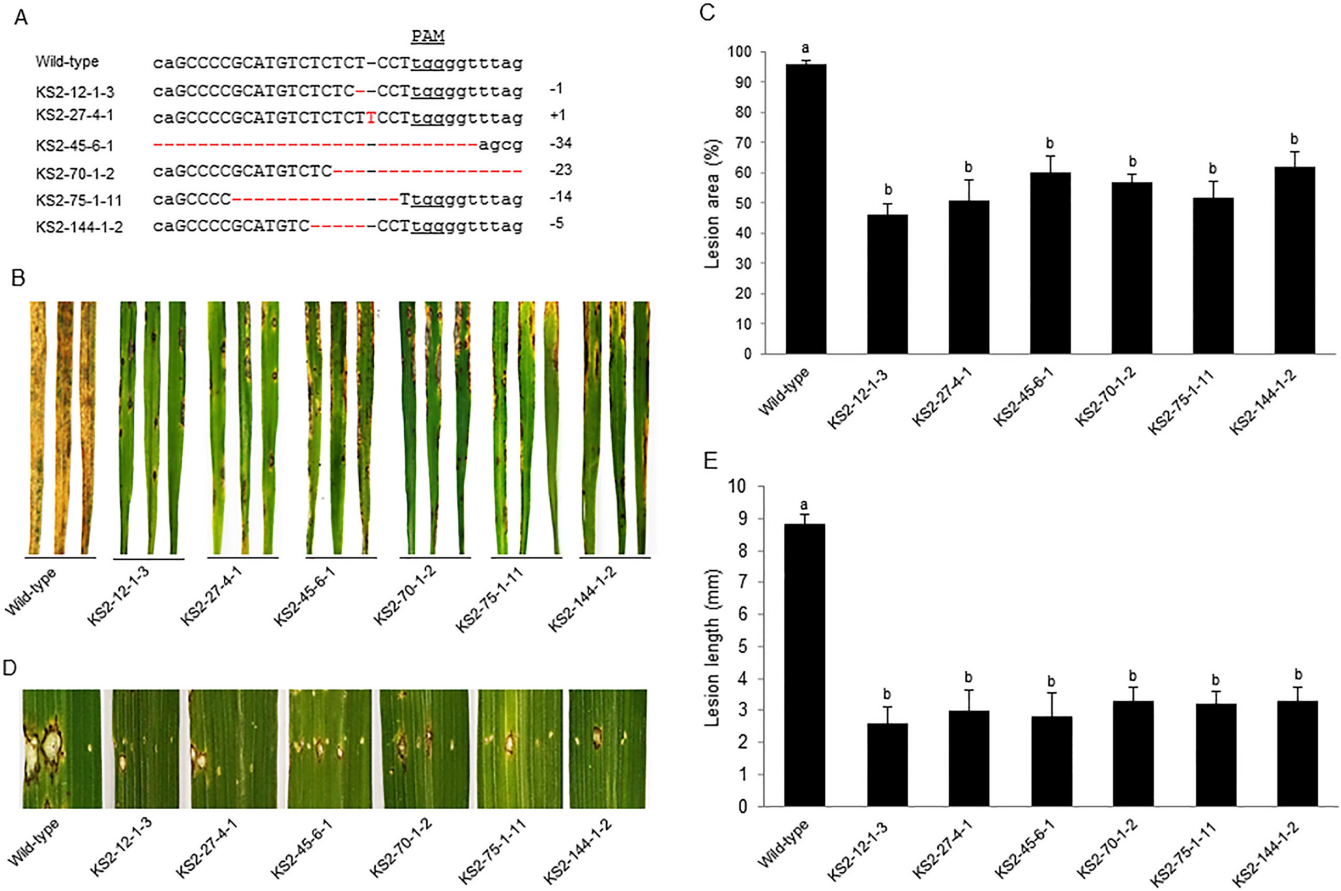
Identification of blast resistance in homozygous mutant rice lines. (A) Nucleotide sequences of the target site in the 6 homozygous T_2_ mutant rice lines used for pathogen inoculation. The recovered mutated alleles are shown below the wild-type sequence. The target site nucleotides are indicated using black capital letters and black dashes. The PAM site nucleotides are underlined. Red dashes indicate the deleted nucleotides. Red capital letters indicate the inserted nucleotides. The numbers on the right indicate the type of mutation and the number of nucleotides involved. “−” and “+” indicate the deletion and insertion of the indicated number of nucleotides, respectively. (B) The blast resistance phenotypes of the mutant rice lines and wild-type plants at the seedling stage. Leaves were detached from the inoculated plants at 7 dpi for photography. The experiments were repeated three times with similar results. (C) Histograms showing the average area of lesions formed on the third leaves of 10 plants for each line. The values marked with different letters are significantly different (P < 0.01, Student’s t-test). (D) Blast resistance phenotypes of the mutant rice lines and the wild-type plants at the tillering stage. Leaves were detached from the inoculated leaves of plants at 7 dpi for photography. The experiments were repeated three times with similar results. (E) Histograms showing the average length of lesions formed on the inoculated leaves of five tillerings for each line. The values marked with different letters are significantly different (P < 0.01, Student’s t-test).

To characterize the blast resistance phenotype of the rice mutants, 6 homozygous mutant T_2_ lines (Fig 3A) with different types of allelic mutations were inoculated with the fungal pathogen *M*. *oryzae* isolate 06-47-6 at the seedling stage. The leaves of wild-type plants nearly died due to pathogen infection, likely because the pathogenicity of isolate 06-47-6 was very strong, and the wild-type variety was highly susceptible (Fig 3B). Nevertheless, the lesion areas formed by pathogen infection were significantly decreased in all mutant rice lines compared with wild-type plants (Fig 3B). The differences were further evaluated by quantification of the lesion areas and significance analysis using Student’s t-test (Fig 3C), which indicated that the mutant rice lines enhanced rice blast resistance. Similarly, lesion lengths formed by pathogen infection were also decreased in the mutant rice lines compared with the wild-type plants at the tillering stage (Fig 3D), and significant difference analysis of quantitative lesion length revealed that all mutant rice lines were significantly different from wild-type plants (Fig 3E). These results indicated that C-ERF922-induced frame shifts in the *OsERF922* gene enhanced resistance to *M*. *oryzae* in the rice mutants because OsERF922 negatively regulates the blast resistance of rice [18].

### The main agronomic traits were not altered in C-ERF922-induced rice mutants

To determine whether mutations in the *OsERF922* gene affect agronomic traits, we characterized all 6 homozygous T_2_ mutant lines (Fig 3A) by measuring their plant height, flag leaf length and width, the number of productive panicles, panicle length, the number of grains per panicle, seed setting rate, and thousand seed weight. Student’s t-test revealed that none of the 6 T_2_ mutant lines differed significantly from wild-type plants under normal growth conditions with regard to the agronomic traits investigated (Table 3).

**Table 3:** Analysis of the agronomic traits of 6 homozygous T_2_ mutant lines.

Mutant lines	Plant height (cm)	Flag leaf length (cm)	Flag leaf width (mm)	No. of productive panicles	Panicle length (cm)	No. of grainsper panicle	Seed setting rate (%)	Thousand seed weight (g)
**WT**	59.8±1.9^a^	26.2±3.2^a^	13.6±0.5^a^	9.0±1.0^a^	12.4±1.0^a^	69.6±3.2^a^	90.0±1.1^a^	26.3±0.3^a^
**KS2-12-1-3**	60.1±1.5^a^	26.8±3.3^a^	13.4±0.5^a^	9.2±1.3^a^	12.9±1.4^a^	71.2±2.8^a^	90.2±1.5^a^	26.2±0.3^a^
**KS2-27-4-1**	59.8±2.4^a^	27.2±2.9^a^	13.6±1.1^a^	9.4±1.5^a^	12.6±0.7^a^	69.2±3.0^a^	89.0±0.8^a^	26.5±0.3^a^
**KS2-45-6-1**	60.8±1.6^a^	25.4±1.9^a^	13.8±0.8^a^	9.2±1.4^a^	12.7±1.6^a^	67.8±3.1^a^	89.1±1.9^a^	26.1±0.1^a^
**KS2-70-1-2**	60.0±2.1^a^	25.8±1.6^a^	13.6±1.2^a^	9.4±1.6^a^	13.0±0.5^a^	68.8±3.7^a^	88.4±0.5^a^	26.2±0.2^a^
**KS2-75-1-11**	59.2±1.9^a^	26.4±3.6^a^	13.8±0.9^a^	9.2±1.5^a^	12.3±0.9^a^	71.8±2.8^a^	89.4±0.9^a^	26.4±0.3^a^
**KS2-144-1-2**	59.6±1.7^a^	25.2±3.3^a^	13.6±1.3^a^	9.4±1.7^a^	12.5±0.5^a^	69.0±2.4^a^	89.8±1.1^a^	26.3±0.2^a^

The results are shown for five mutant plants of each mutant line and are represented as the mean ± SE. The values marked with the same letter (a) are non-significantly different (P < 0.05, Student’s t-test).

### The mutagenic frequency and mutagenic frequency of homozygous plants were increased by targeting multiple sites within *OsERF922*

To examine whether the mutagenic frequency could be increased by targeting multiple sites within one gene, we designed Cas9/two-target-sgRNAs (C-ERF922S1S2) and Cas9/three-target-sgRNAs (C-ERF922S1S2S3) to target two sites (ERF922-S1 and ERF922-S2, Fig 1A and S1 Fig) and three sites (ERF922-S1, ERF922-S2 and ERF922-S3; Fig 1A and S1 Fig) within the *OsERF922* gene in rice, respectively. Higher overall mutagenic frequencies and mutagenic frequencies of homozygous plants are of value because they facilitate the generation of homozygous mutants in the T_0_ generation for crop improvement. The C-ERF922S1S2- and C-ERF922S1S2S3-expressing constructs (pC-ERF922S1S2 and pC-ERF922S1S2S3, S2 Fig) were generated based on CRISPR/Cas9 multi-targeting vector described by Ma et al. [38] and were used to transform the rice variety Kuiku131 by *Agrobacterium*-mediated transformation. We obtained 30 positive transgenic (T_0_) plants for the analysis of mutations for each transformation and detected 21 (70.0%) and 27 (90.0%) mutant plants, respectively (Table 4, S2 and S3 Tables). Direct Sanger-sequencing of the target-containing amplicons followed by decoding with the DSD method [66, 67] showed that among the C-ERF922S1S2-induced mutant plants were 19 (63.3%) plants harboring mutations at both target sites (Table 4 and S2 Table). Furthermore, 47.6% (10/21, Table 5) of the mutants were homozygotes. In addition, among C-ERF922S1S2S3-induced mutant plants, all 27 (90.0%) plants harbored mutations at all three target sites (Table 4 and S3 Table), and 40.7% (11/27, Table 5) of the mutants were homozygotes. These results demonstrated that the mutagenic frequencies increased when targeting more sites within one gene, and Cas9/two-target-sgRNAs resulted in the highest mutagenic frequency in homozygotes (Tables 1 and 5).

**Table 4:** Targeting multiple sites in rice using CRISPR/Cas9 and the number of plants with mutations at single, double and triple target sites.

		No. of plants harboring mutations at target sites			
Transformant	No. of tested plants	ERF922-S1	ERF922-S2	ERF922-S3	All*
**pC-ERF922S1S2**	30	21(70.0%)	19(63.3%)		19(63.3%)
**pC-ERF922S1S2S3**	30	27(90.0%)	27(90.0%)	27(90.0%)	27(90.0%)

* Based on the number of plants with mutations for all combinations of target sites.

**Table 5:** Targeting multiple sites in rice using CRISPR/Cas9 and the mutant genotype ratios at the target sites.

	ERF922-S1 (%) ^#^			ERF922-S2 (%) ^#^			ERF922-S3 (%) ^#^			
Transformant	Hetero	Bi	Homo	Hetero	Bi	Homo	Hetero	Bi	Homo	All Homo *
**pC-ERF922S1S2**	14.3(3/21)	28.6(6/21)	57.1(12/21)	15.8(3/19)	26.3(5/19)	57.9(11/19)				47.6(10/21)
**pC-ERF922S1S2S3**	3.8(1/27)	37.0(10/27)	59.3(16/27)	3.8(1/27)	44.4(12/27)	51.8(14/27)	3.8(1/27)	48.1(13/27)	48.1(13/27)	40.7(11/27)

^**#**^ Based on the number of mutant genotypes out of the total number of mutant genotypes at each the target site.

* Based on the number of homozygote genotypes out of the total number mutant genotypes at all target sites. Hetero: Heterozygote; Bi: Bi-allele; Homo: Homozygote.

## Discussion

Genome editing using SSNs provides an opportunity for crop improvement. Thus far, SSNs have been used to improve a variety of important crops, such as rice [56, 60], wheat [47], maize [55], soybean [58] and potato [59], by creating specific gene knockouts. However, examples of crop improvement via the creation of novel genotypes, agronomic traits or disease resistance remain limited. The first example was using ZFNs to target the maize *IPK1* gene, which resulted in reduced levels of phytate—an anti-nutritional component of feed grains—and reduced phosphate pollution in waste streams from cattle-feeding operations [55]. In rice, the disease-susceptibility gene and the sucrose efflux transporter gene *OsSWEET14*, which aids in pathogen survival and virulence, was mutated by TALENs to produce disease-resistant rice with normal phenotypes [56]; moreover, using TALENs to target the *OsBADH2* gene produced a generation of fragrant rice that contain 2-acetyl-1-pyrroline (2AP), a major fragrance compound [60]. In addition, knocking out all three MILDEW-RESISTANCE LOCUS (MLO) alleles in bread wheat using one pair of TALENs resulted in the creation of stable mutant lines exhibiting broad-spectrum resistance to powdery mildew [47]. In the present study, we used C-ERF922, C-ERF922S1S2 and C-ERF922S1S2S3 to knockout *OsERF922* and achieved 42.0%, 70.0% and 90.0% recovery of C-ERF922-, C-ERF922S1S2- and C-ERF922S1S2S3-induced mutant plants, respectively, in T_0_ transgenic plants; all of the allele mutations were transmitted to the T_1_ and T_2_ generations. We obtained more than 20 mutant plants that harbor the desired modification in *OsERF922* but not containing the transgene, which was eliminated via segregation in the T_1_ and T_2_ generations. Inoculation with *M*. *oryzae* revealed that blast resistance in the T_2_ homozygous mutant lines tested was significantly enhanced compared with that of wild-type plants at both the seedling and tillering stages. In addition, we showed that there was no significant difference between T_2_ homozygous mutant lines and wild-type plants with respect to the agronomic traits, such as plant height, flag leaf length and width, the number of productive panicles, panicle length, the number of grains per panicle, seed setting rate, and thousand seed weight. This study provides a successful example of improving rice blast resistance using CRISPR/Cas9 technology.

The C-ERF922-induced mutagenic frequency of T_0_ plants in this study was 42.0%, similar to those previously reported for CRISPR/Cas9-induced mutations in rice [31, 35, 38, 39]. In addition, the genotypes of these T_0_ mutant plants were primarily bi-allelic (76.1%) and homozygotic (14.3%) (Table 1), which was also similar to previous observations [29–33, 35–38, 71]. This phenomenon is in stark contrast to that of TALEN-induced mutant genotypes, wherein the heterozygous mutants were more frequent than bi-allele mutants in T_0_ plants [60, 64, 72, 73]. This difference might be caused by the different target site cleavage efficiencies of CRISPR/Cas9 and TALENs.

Multiple mutations were detected at the target site in the T_0_ mutant plant KS2-12 (Fig 1C). The presence of chimeric mutations in a single mutant plant may result from delayed cleavage in the primary embryogenic cell. This phenomenon has been reported in rice [29, 34, 35, 38], *Arabidopsis* [74], wheat [47], tomato [49] and maize [42]. In addition, the segregation ratios observed for the T_1_ plants derived from KS2-12 were not Mendelian, probably because the chimeric mutations were restricted to somatic cells that did not participate in the production of gametes. Furthermore, the segregation ratios found in T_1_ plants obtained from the bi-allelic mutant plant KS2-45 did not conform to a Mendelian ratio, but the T_2_ plants derived from the same bi-allelic mutant plant did (Table 2), indicating that the number of T_1_ plants was smaller or that homozygous mutation (11-bp deletion) may be induced through detrimental mutations caused by T-DNA insertion, which has less chance of survival compared with the other types of mutants. Notably, several novel mutations were detected in the T_2_ offspring of KS2-44-1 (S5 Fig). This could be explained by the fact that KS2-44-1 was a heterozygote in which the C-ERF922 construct remained active and continually cleaved the target site in T_2_ plants, resulting in new mutations. However, we did not detect new mutations in the T_1_ offspring of KS2-44, probably due to the existence of only 3 T_1_ plants (Table 2).

Both CRISPR/Cas9 and TALENs are effective tools for gene modification; however, each has specific advantages and limitations. Compared with TALENs, CRISPR/Cas9-expressing vectors are much easier to construct and can be competed in just two or three days [38, 75], whereas the construction of TALENs typically requires over seven days [76–78]. Furthermore, CRISPR/Cas9 induces a much higher mutation rate than TALENs. For example, the frequency of CRISPR/Cas9-targeted mutagenesis ranged from 21.1% to 66.7% (average 44.4%) for 11 rice genes [35]. Likewise, Ma and colleagues recently reported that the average mutation rate was 85.4% for CRISPR/Cas9-based editing of 46 target sites in rice [38]. However, the frequency of TALEN-targeted mutagenesis ranged from 0–30% overall [47, 64, 73]. Nevertheless, the requirement for a PAM (-NGG) sequence and the possibility of off-target effects are limitations of CRISPR/Cas9 system. For example, previous studies have demonstrated that off-target effects were common at the level of one nucleotide mismatch in plant species [37, 50], fish [79] and human cells [80, 81]. In contrast, off-target effects were extremely rare in the event of one nucleotide mismatch for the TALENs-editing system [82, 83]. The present study indicates that the CRISPR/Cas9 system is indeed a powerful tool for crop improvement via site-specific genome editing.

## Supporting Information

S1 Fig Schematic of the *OsERF922* gene structure and the ERF922-S1 and ERF922-S3 target sites. (TIF)

S2 Fig Schematic diagram of the pC-ERF922S1S2 and pC-ERF922S1S2S3 constructs for expressing the CRISPR/Cas9 proteins C-ERF922S1S2 and C-ERF922S1S2S3. (TIF)

S3 Fig Nucleotide sequences of the C-ERF922-induced mutations at the target site in the rice protoplasts. (TIF)

S4 Fig Nucleotide sequences at the target site in C-ERF922-induced T_0_ mutant plants. (TIF)

S5 Fig Nucleotide sequences of the C-ERF922-induced novel mutations in the offspring of KS2-44-1. (TIF)

S1 Table Primers used in this study. (PDF)

S2 Table Nucleotide sequences at the target sites in C-ERF922S1S2-induced T_0_ mutant rice plants. (PDF)

S3 Table Nucleotide sequences at the target sites in C-ERF922S1S2S3-induced T_0_ mutant rice plants. (PDF)
