# Supplementary figures and images for: Enhanced Rice Blast Resistance by CRISPR/Cas9-Targeted Mutagenesis of the ERF Transcription Factor Gene OsERF922
Source: PLoS One. 2016 Apr 26;11(4):e0154027. doi: 10.1371/journal.pone.0154027 (PMC4846023; doi:10.1371/journal.pone.0154027)

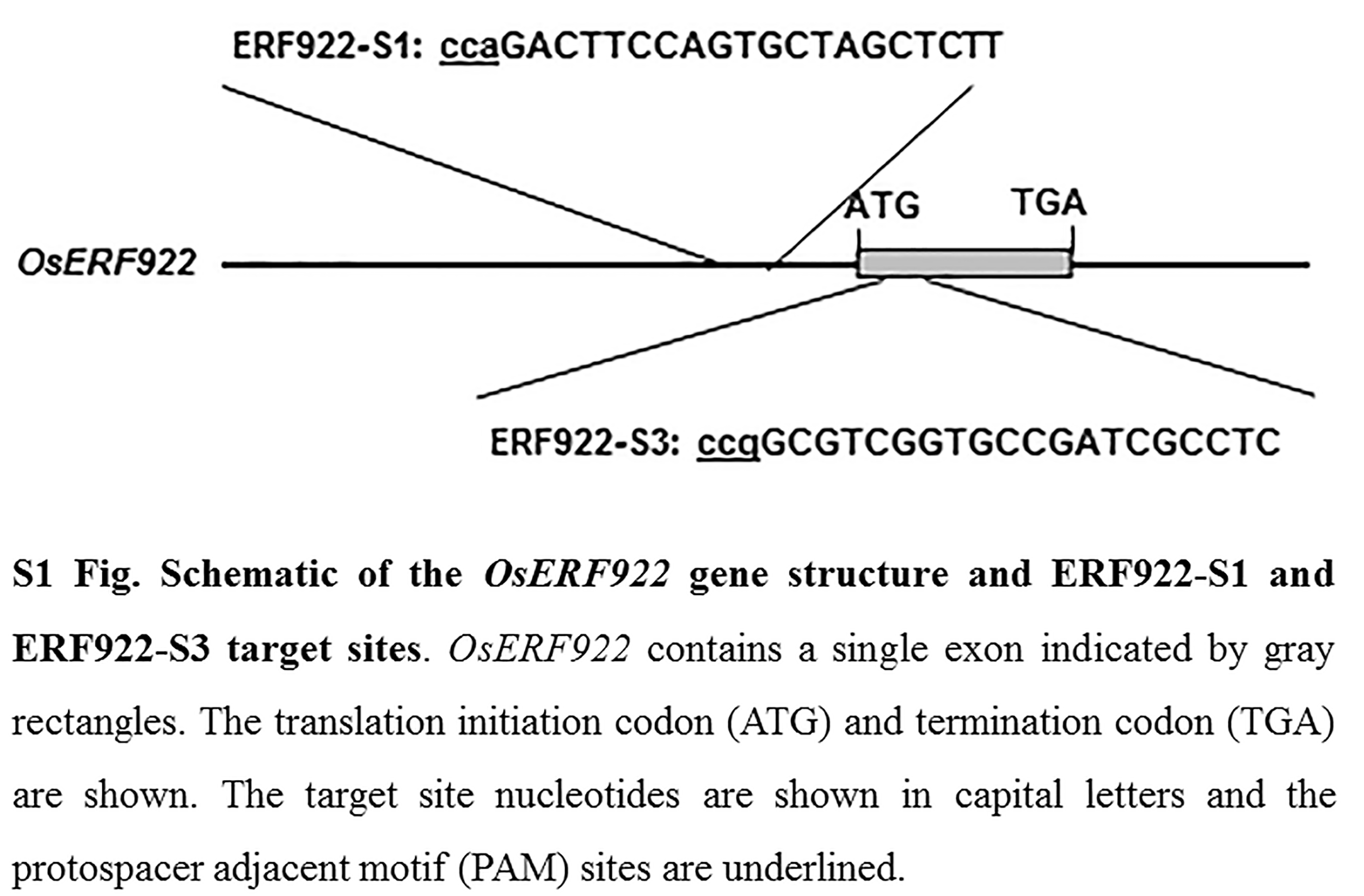

Supplement: S1 Fig — (TIF) [file pone.0154027.s001.tif]

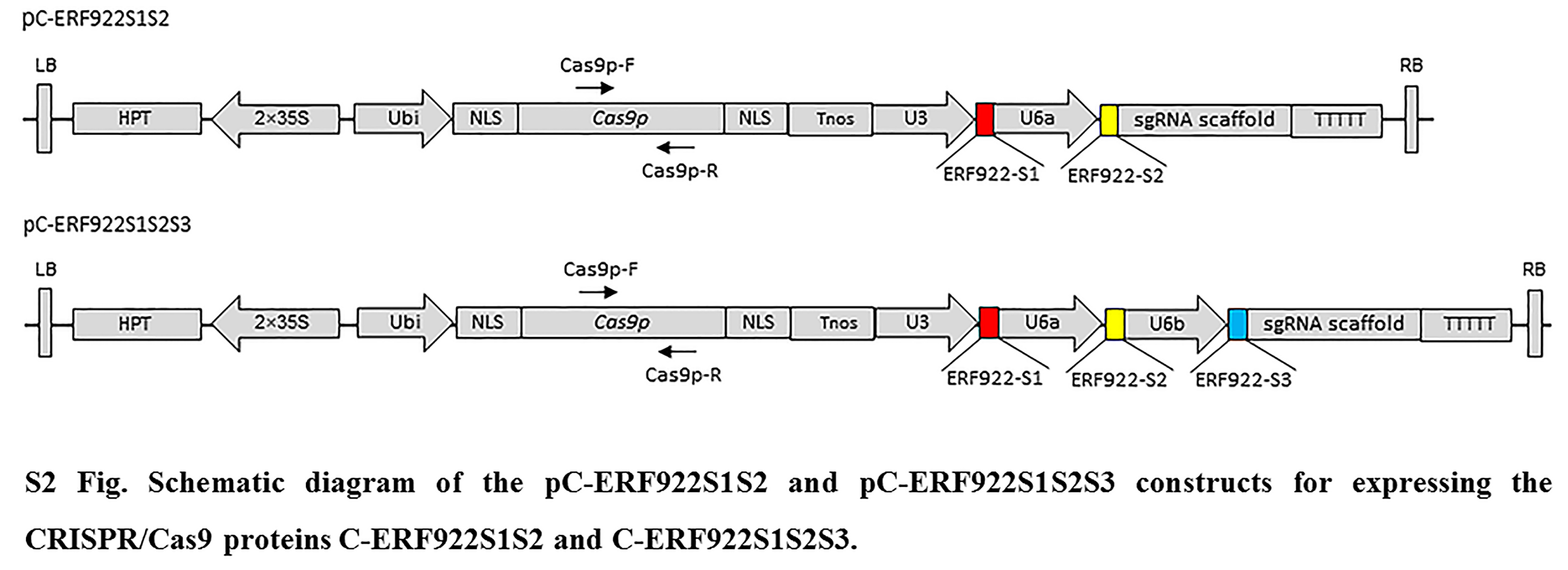

Supplement: S2 Fig — (TIF) [file pone.0154027.s002.tif]

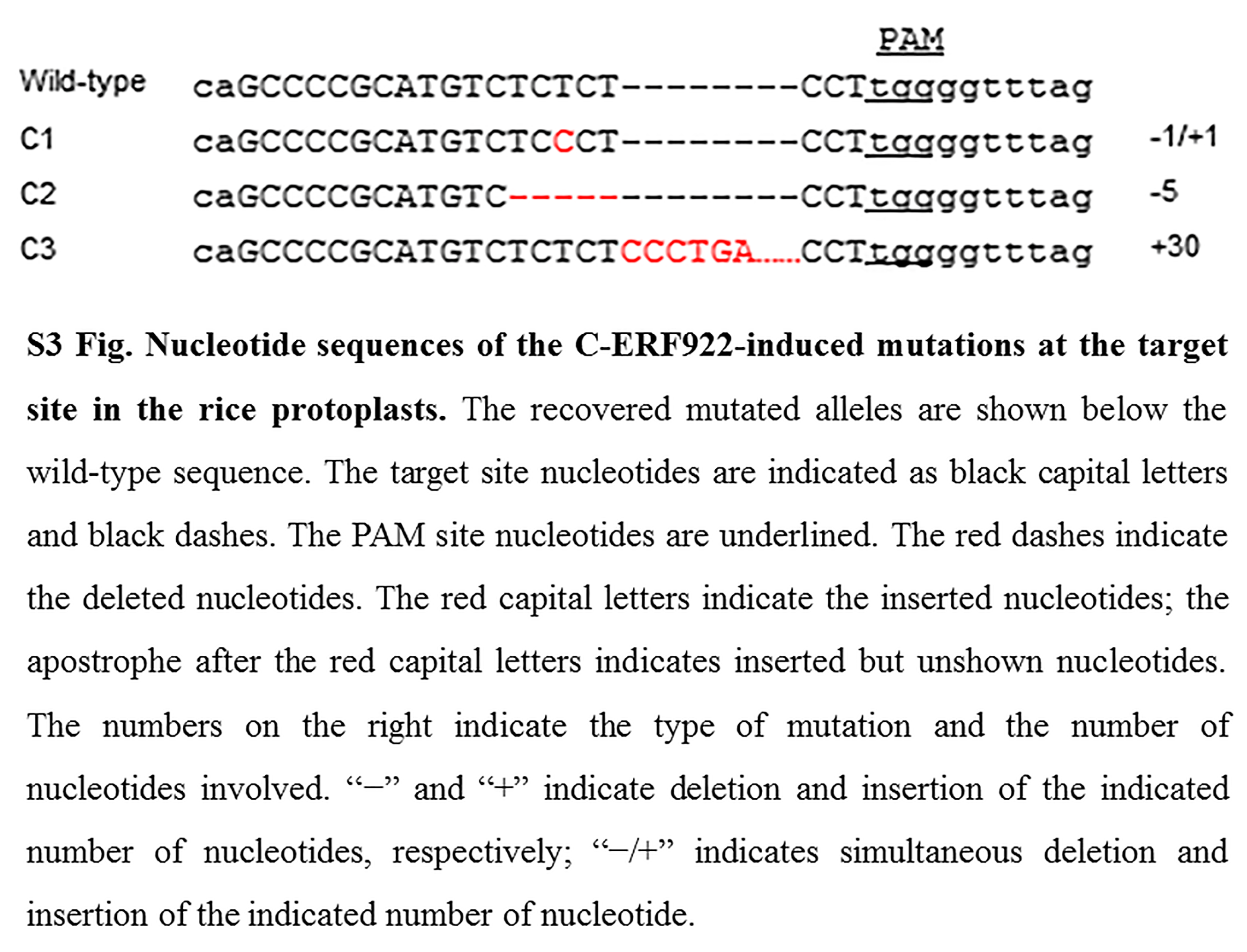

Supplement: S3 Fig — (TIF) [file pone.0154027.s003.tif]

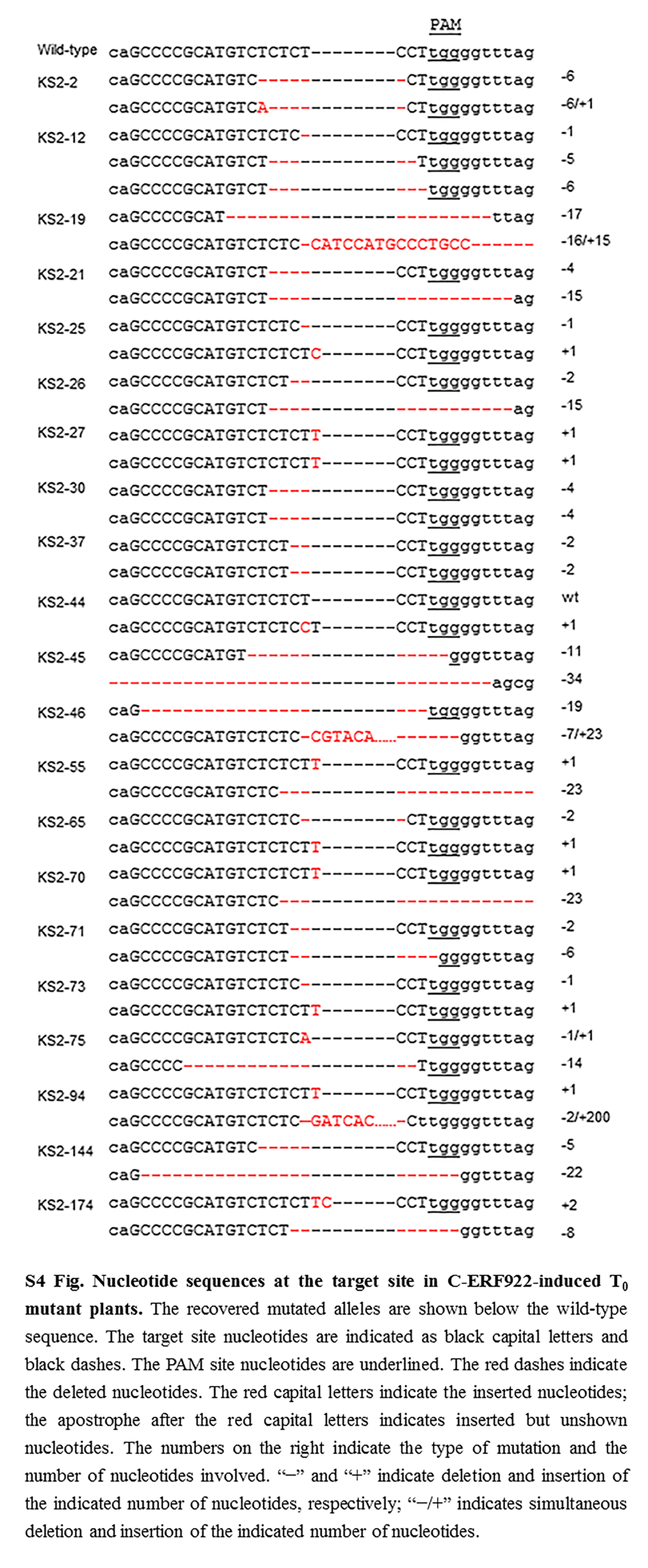

Supplement: S4 Fig — (TIF) [file pone.0154027.s004.tif]

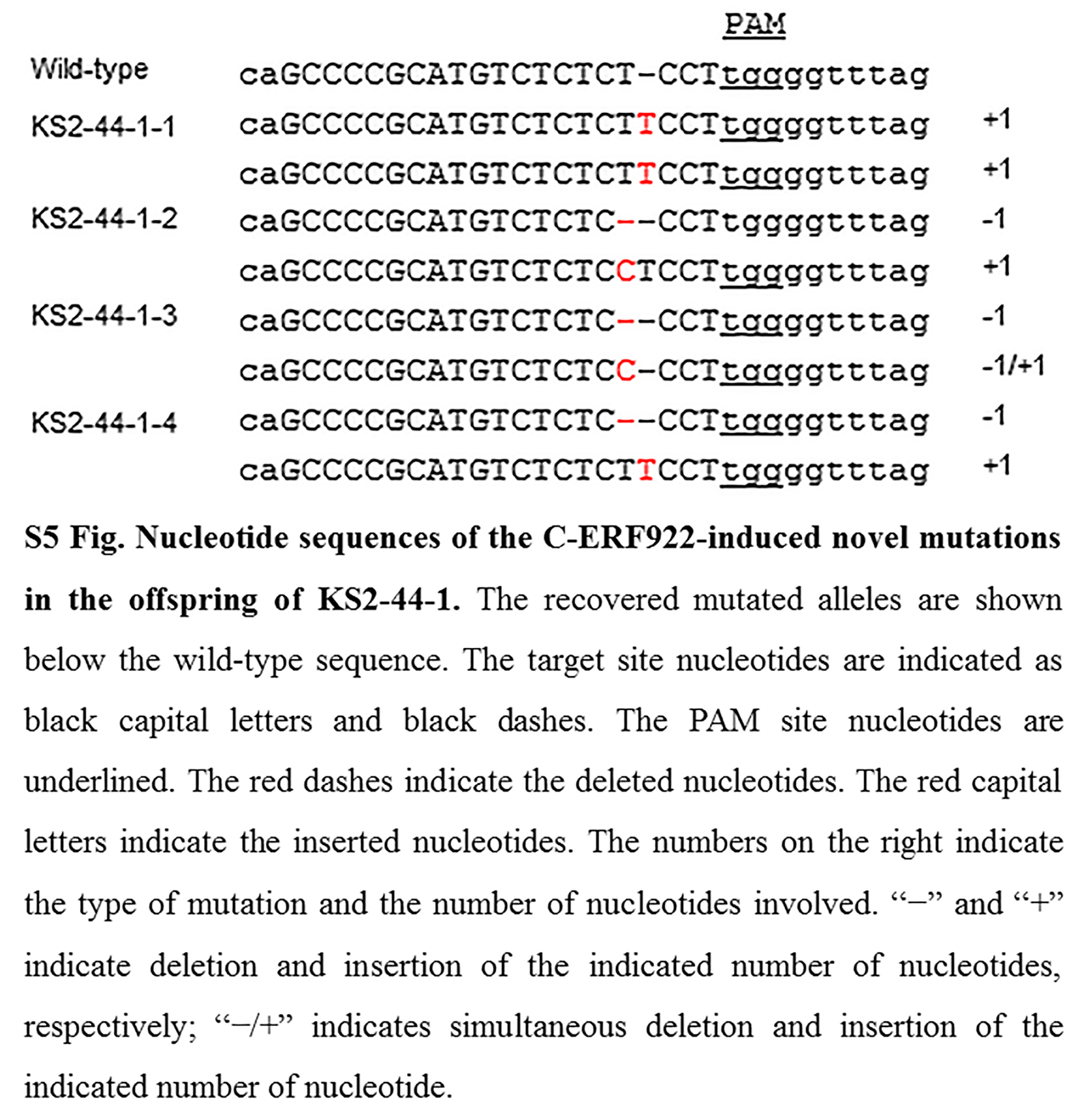

Supplement: S5 Fig — (TIF) [file pone.0154027.s005.tif]
