# Supplementary material for: Enhanced Rice Blast Resistance by CRISPR/Cas9-Targeted Mutagenesis of the ERF Transcription Factor Gene OsERF922
Source: PLoS One. 2016 Apr 26;11(4):e0154027. doi: 10.1371/journal.pone.0154027 (PMC4846023; doi:10.1371/journal.pone.0154027)
Supplement: S1 Table — (PDF) [file pone.0154027.s006.pdf]

S1 Table. Primers used in this study

Primers	Primer sequence (5'→3')	For experiment
E922-FS1	GGCAAGAGCTAGCACTGGAAGTC	Vector construct
E922-RS1	AAACGACTTCCAGTGCTAGCTCT	
E922-FS2	GCCGCCCCGCATGTCTCTCTCCT	Vector construct
E922-RS2	AAACAGGAGAGAGACATGCGGGG	
E922-FS3	GTTGAGGCGATCGGCACCGACGC	Vector construct
E922-RS3	AAACGCGTCGGTGCCGATCGCCT	
U-F	CTCCGTTTTACCTGTGGAATCG	Vector construct
gR-R	CGGAGGAAAATTCCATCCAC	
Pps-GGL	TTCAGAGGTCTCTCTCGACTAGTATGGAATCGGCAGCAAAGG	Vector construct
Pgs-GG2	AGCGTGGGTCTCGACCGACGCGTATCCATCCACTCCAAGCTC	
Pps-GG2	AGCGTGGGTCTCGTCAGGGTCCATCCACTCCAAGCTC	
Pgs-GG3	TTCAGAGGTCTCTCTGACACTGGAATCGGCAGCAAAGG	
Pps-GG3	AGCGTGGGTCTCGTCTTCACTCCATCCACTCCAAGCTC	
Pgs-GGR	TTCAGAGGTCTCTAAGACTTTGGAATCGGCAGCAAAGG	
E922-KF	AGCTCTTCCTCGGGTTCAAA	Amplification of fragments across ERF922-S2
E922-KR	AGGCGATCGGCACCGACGC	
E922P-2F	CCGAGGAGGTTGCGAAATCA	Amplification of fragments across all target sites
E922-KR2	ACAGAGACACGTCCACGCGT	
Cas9p-F	TTCGACCAGTCCAAGAACGG	*Cas9* detection
Cas9p-R	CTTGACCTTGGTGAGCTCGT	

The restriction enzyme site *Bsa*I was underlined.
