# Supplementary material for: Enhanced Rice Blast Resistance by CRISPR/Cas9-Targeted Mutagenesis of the ERF Transcription Factor Gene OsERF922
Source: PLoS One. 2016 Apr 26;11(4):e0154027. doi: 10.1371/journal.pone.0154027 (PMC4846023; doi:10.1371/journal.pone.0154027)
Supplement: S2 Table — (PDF) [file pone.0154027.s007.pdf]

S2 Table. Nucleotide sequences at the target sites in C-ERF922S1S2-induced T_0 mutant rice plants

Mutant plants	Sequence (5'-3')	Mutation type
	S1 S2	
wild-type	ttccaGAC--TTCCAGTGCTAGCTCTTcct..........acaGCCCCGCATGTCTCTCT-CCTtgggg	wt
KS12-31	-----------------------------..........acaGCCCCGCATG------------gggg	S1: -80, S2: -11
	-----------------------------..........acaGCCCCGCATG------------gggg	S1: -80, S2: -11
KS12-34	ttccaGACT-TTCCAGTGCTAGCTCTTcct..........acaGCCCCGCATGTCTCT---CCTtgggg	S1: +1, S2: -2
	ttccaGACT-TTCCAGTGCTAGCTCTTcct..........acaGCCCCGCATGTCTCT---CCTtgggg	S1: +1, S2: -2
KS12-36	ttccaGACT-TTCCAGTGCTAGCTCTTcct..........acaGCCC--------------------gg	S1: +1, S2: -19
	ttccaGACT-TTCCAGTGCTAGCTCTTcct..........TTGGCCTGGAGCCCGGACATTTTGCGC......	S1: +1, S2: -42/+41
KS12-48	ttcca----------GTGCTAGCTCTTcct..........acaGCCCCGCATGTCTCTC-----tgggg	S1: -8, S2: -4
	ttcca----------GTGCTAGCTCTTcct..........acaGCCCCGCATGTCTCTC-----tgggg	S1: -8, S2: -4
KS12-100	ttccaGAC--TTC--	S1S2: -195
	ttccaGAC--TTC--	S1S2: -195
KS12-101	ttccaGAC--TTCCAGTGCTAGCTCTTcct..........acaGCCCCGCATGTCTCTCT-CCTtgggg	wt
	ttccaGACT-TTCCAGTGCTAGCTCTTcct..........acaGCCCCGCATGTCTCTCT-----gggg	S1: +1, S2: -4
KS12-114	ttccaGA--------------GCTCTTcct..........acaGCCCCGCATGTCTCTCT-CCTtgggg	S1: -12, S2: wt
	ttccaGA--------------GCTCTTcct..........acaGCCCCGCATGTCTCTCT-CCTtgggg	S1: -12, S2: wt
KS12-124	ttccaGAC--T--CCTtgggg	S1S2: -168
	ttccaGAC--T--CCTtgggg	S1S2: -168
KS12-125	ttccaGAC------GTGCTAGCTCTTcct..........acaGCCCCGCATGTCTCTC-----Ttgggg	S1: -5, S2: -4
	ttccaGACA-TTCCAGTGCTAGCTC------.........acaGCCCCGCATGT----AA-CCTtgggg	S1: -6/+1, S2: -6/+2

Continued the table

KS12-130	ttccaGAC------AGTGCTAGCTCTTcct...........acaGCCCCGCATGTCTCTCTACCTtgggg	S1: -4, S2: +1
	ttccaGAC-------GTGCTAGCTCTTcct...........acaGCCCCGCATGTCTCTCTTCCTtgggg	S1: -5, S2: +1
KS12-162	ttccaGACT-TTCCAGTGCTAGCTCTTcct...........acaGCCCCGCATGTC----T-CCTtgggg	S1: +1, S2: -4
	ttccaGACTTTTCCAGTGCTAGCTCTTcct...........acaGCCCCGCATGTC----T-CCTtgggg	S1: +2, S2: -4
KS12-163	ttccaGAC--T--CCTtgggg	S1S2: -168
	ttccaGAC--T--CCTtgggg	S1S2: -168
KS12-164	ttccaGAC--T--CCTtgggg	S1S2: -168
	ttccaGAC--T--CCTtgggg	S1S2: -168
KS12-175	ttccaGAC-TTTCCAGTGCTAGCTCTTcct...........acaGCCCCGCATGTC---------tgggg	S1: +1, S2: -8
	ttccaGAC-TTTCCAGTGCTAGCTCTTcct...........acaGCCCCGCATGTC---------tgggg	S1: +1, S2: -8
KS12-181	ttccaGAC---TCCAGTGCTAGCTCTTcct...........acaGCCCCGCATGTCTCTC-----tgggg	S1: -1, S2: -4
	ttccaGAC----CCAGTGCTAGCTCTTcct...........acaGCCCCGCATGTCTCTCT-CCTtgggg	S1: -2, S2: wt
KS12-182	ttccaGAC---CCTtgggg	S1S2: -169
	ttccaGAC---CCTtgggg	S1S2: -169
KS12-183	ttccaGAC--TTCCAGTGCTAGCTCTTcct...........acaGCCCCGCATGTCTCTCT-CCTtgggg	wt
	ttccaGAC---CCTtgggg	S1S2: -169
KS12-185	ttccaG---	S1S2: -203
	ttccaG---	S1S2: -203
KS12-213	ttccaGAC--TTCCAGTGCTAGCTCTTcct...........acaGCCCCGCATGTCTCTCT-CCTtgggg	wt
	ttccaGA--------------GCTCTTcct...........acaGCCCCGCATGTCTCTCT-CCTtgggg	S1: -12, S2: wt
KS12-214	ttccaGAC---TCCAGTGCTAGCTCTTcct...........acaGCCCCGCATGTCTCTCTTCCTtgggg	S1: -1, S2: +1
	ttccaGAC----CCAGTGCTAGCTCTTcct...........acaGCCCCGCATGTCTCTC-----tgggg	S1: -2, S2: -4
KS12-231	ttccaGACA-TTCCAGTGCTAGCTCTTcct...........acaGCCCCGCATGTCTCTCTACCTtgggg	S1: +1, S2: +1
	ttccaGAC-------GTGCTAGCTCTTcct...........acaGCCCCGCATG-----------tgggg	S1: -5, S2: -10

The mutated alleles are shown below the wild-type sequence. The distance between ERF922-S1 and ERF922-S2 is 135-bp (the black apostrophe indicates nucleotides are not shown in.). The target sites nucleotides are shown in black capital letters and black dashes. The PAM site nucleotides are underlined. The red dashes indicate the deleted nucleotides. The red capital letters indicate inserted nucleotides and the apostrophe followed to red capital letters indicates inserted nucleotides are not shown in. "−" and "+" indicate deletion and insertion of the indicated number of nucleotides, respectively "−/+" indicates simultaneous deletion and insertion of the indicated number of nucleotides. S1: ERF922-S1; S2: ERF922-S2; S1S2: deletion between ERF922-S1 and ERF922-S2; wt: wild-type sequence.
