# Supplementary material for: Enhanced Rice Blast Resistance by CRISPR/Cas9-Targeted Mutagenesis of the ERF Transcription Factor Gene OsERF922
Source: PLoS One. 2016 Apr 26;11(4):e0154027. doi: 10.1371/journal.pone.0154027 (PMC4846023; doi:10.1371/journal.pone.0154027)
Supplement: S3 Table — (PDF) [file pone.0154027.s008.pdf]

S3 Table. Nucleotide sequences at the target sites in C-ERF922S1S2S3-induced T_0 mutant rice plants

Mutant plants	Sequence (5'-3')	Mutation type
	S1 S2 S3	
wild-type	ttccaGAC-TTCCAGTGCTAGCTCTTcct...........acaGCCCCGCATGTCTCTCT-CCTtgggg...........ccgGCG-TCGGTGCCGATCGCCTCgcc	wt
KS123-6	ttccaGAC-T--CGGTGCCGATCGCCTCgcc	S1S3: -195
	ttccaGAC-T--CGGTGCCGATCGCCTCgcc	S1S3: -195
KS123-8	ttccaGAC-T--CGGTGCCGATCGCCTCgcc	S1S3: -195
	ttccaGAC-T--CGGTGCCGATCGCCTCgcc	S1S3: -195
KS123-13	ttccaGACATTCCAGTGCTAGCTCTTcct...........acaGCCCCGCATGTCTCTC--CCTtgggg...........ccgGCGTTCGGTGCCGATCGCCTCgcc	S1: +1, S2: -1, S3: +1
	tt--------TCCAGTGCTAGCTCTTcct...........acaGCCCCGCATGTCTCTC--CCTtgggg...........ccgG---TCGGTGCCGATCGCCTCgcc	S1: -7, S2: -1, S3: -2
KS123-20	ttccaGAC-T--CCTtgggg...........ccgGCG---GGTGCCGATCGCCTCgcc	S1S2: -168, S3: -2
	ttccaGAC-T--CCTtgggg...........ccgGCGCTCGGTGCCGATCGCCTCgcc	S1S2: -168, S3: +1
KS123-25	ttccaGAC----------------------........acaGCCCCGCATGTCTCTCCGCCTtgggg...........ccgGCG-T--------ATCGCCTCgcc	S1: -42, S2: -1/+2, S3: -8
	ttccaGAC----------------------........acaGCCCCGCATGTCTCTCCGCCTtgggg...........ccgGCG-T--------ATCGCCTCgcc	S1: -42, S2: -1/+2, S3: -8
KS123-33	ttccaGAC-------------CTCTTcct...........acaGCCCCGCATGTCTCTCT--------------------TCGGTGCCGATCGCCTCgcc	S1: -12, S2S3: -27
	ttccaGACATTCCAGTGCTAGCTCTTcct...........acaGCCCCGCATGTCTCT---GGACATgg...........ccgGCGCTCGGTGCCGATCGCCTCgcc	S1: +1, S2: -8/+6, S3: +1
KS123-35	ttccaGAC---CCAGTGCTAGCTCTTcct...........acaGCC--GATCGCCTCgcc	S1: -2, S2S3: -49
	ttccaGAC--TCCAGTGCTAGCTCTTcct...........acaGCCCCGCATGTCTCTC--CCTtgggg...........ccgGCGATCGGTGCCGATCGCCTCgcc	S1: -1, S2: -1, S3: +1
KS123-37	ttccaGACTTTCCAGTGCTAGCTCTTcct...........acaGCCCCGCATGTCTCTCTTCCTtgggg...........ccgGCGATCGGTGCCGATCGCCTCgcc	S1: +1, S2: +1, S3: +1
	ttccaGACTTTCCAGTGCTAGCTCTTcct...........acaGCCCCGCATGTCTCTCTTCCTtgggg...........ccgGCGATCGGTGCCGATCGCCTCgcc	S1: +1, S2: +1, S3: +1
KS123-38	ttccaGACATTCCAGTGCTAGCTCTTcct...........acaGCCCCGCATGTCTCTCT--------------------TCGGTGCCGATCGCCTCgcc	S1: +1, S2S3: -27
	ttcca---GTGCCGATCGCCTCgcc	S1S3: -202
KS123-43	ttccaGAC---CCAGTGCTAGCTCTTcct...........acaGCCCCGCATGTCTCTCT--------------------TCGGTGCCGATCGCCTCgcc	S1: -2, S2S3: -27
	ttccaGAC---CCAGTGCTAGCTCTTcct...........acaGCCCCGCATGTCTCTCT--------------------TCGGTGCCGATCGCCTCgcc	S1: -2, S2S3: -27

Continued the table

KS123-46	ttccaGAC--TCCAGTGCTAGCTCTTcct............acaGCCCCGCATGTCTCTCTTCCTtgggg............ccgGCGATCGGTGCCGATCGCCTCgcc	S1: -1, S2: +1, S3: +1
	ttccaGAC--TCCAGTGCTAGCTCTTcct............acaGCCCCGCATGTCTCTCTTCCTtgggg............ccgGCGATCGGTGCCGATCGCCTCgcc	S1: -1, S2: +1, S3: +1
KS123-47	ttccaGAC--TCCAGTGCTAGCTCTTcct............acaGCCCCGCATGTCTCTC----Ttgggg............ccgGCG----GTGCCGATCGCCTCgcc	S1: -1, S2: -3, S3: -3
	ttccaGACTTTCCAGTGCTAGCTCTTcct............acaGCCCCGCATGTCTCTC--CCTtgggg............ccgGCGTTCGGTGCCGATCGCCTCgcc	S1: +1, S2: -1, S3: +1
KS123-49	ttccaGAC----------------------.........acaGCCCCGCATGTCTCTCT--------------------TCGGTGCCGATCGCCTCgcc	S1: -98, S2S3: -27
	ttccaGAC----------------------.........acaGCCCCGCATGTCTCTCT--------------------TCGGTGCCGATCGCCTCgcc	S1: -98, S2S3: -27
KS123-50	ttccaGAC----------------------.........acaGCCCCGCATGTCTCTCT--------------------TCGGTGCCGATCGCCTCgcc	S1: -98, S2S3: -27
	ttccaGAC----------------------.........acaGCCCCGCATGTCTCTCT--------------------TCGGTGCCGATCGCCTCgcc	S1: -98, S2S3: -27
KS123-60	--GCCGATCGCCTCgcc	S1S3: -221
	ttccaGAC------------GCTCTTcct............acaGCCCCGCATGTCTCTCT--------------------TCGGTGCCGATCGCCTCgcc	S1: -11, S2S3: -27
KS123-71	ttccaGAC-T----GTGCTAGCTCTTcct............acaGCCCCGCATGTCTCTCT---------------------CGGTGCCGATCGCCTCgcc	S1: -4, S2S3: -28
	ttccaGAC-T----GTGCTAGCTCTTcct............acaGCCCCGCATGTCTCTCT---------------------CGGTGCCGATCGCCTCgcc	S1: -4, S2S3: -28
KS123-73	ttccaGAC----------------TTcct............acaGCCCCGCAT---------------gg............ccgGCGTTCGGTGCCGATCGCCTCgcc	S1: -15, S2: -14, S3: +1
	ttccaGAC----------------TTcct............acaGCCCCGCAT---------------gg............ccgGCGTTCGGTGCCGATCGCCTCgcc	S1: -15, S2: -14, S3: +1
KS123-81	ttccaGACTTTCCAGTGCTAGCTCTTcct............acaGCCCCGCATGTCTCTC--CCTtgggg.........------------------------Cgcc	S1: +1, S2: -1, S3: -30
	ttccaGACTTTCCAGTGCTAGCTCTTcct............acaGCCCCGCATGTCTCTCTTCCTtgggg.........ccgGCGTCCGGTGCCGATCGCCTCCgcc	S1: +1, S2: +1, S3: +1
KS123-91	ttccaGA--TTCCAGTGCTAGCTCTTcct............acaGCCCCGCATGTCT-----CCTtgggg............ccgGCG-----TGCCGATCGCCTCgcc	S1: -1, S2: -4, S3: -4
	--------------GTGCTAGCTCTTcct............acaGCCCCGCATGTCTCTCT--------------------TCGGTGCCGATCGCCTCgcc	S1: -16, S2S3: -27
KS123-96	ttccaGAC-TTCC---GTGCCGATCGCCTCgcc	S1S3: -195
	ttccaGAC--TCCAGTGCTAGCTCTTcct............acaGCCCCGCATGTCTCTCTTCCTtgggg............ccgGC--TCGGTGCCGATCGCCTCgcc	S1: -1, S2: +1, S3: -1
KS123-100	ttccaTTT----------------------------GCCCCGCATGTCTCTCTTCCTtgggg............ccgGCGATCGGTGCCGATCGCCTCgcc	S1: -156/+3, S2: +1, S3: +1
	ttccaGAC--TCCAGTGCTAGCTCTTcct............acaGCCCCGCATGTCTCTC---CTtgggg............ccgGCGATCGGTGCCGATCGCCTCgcc	S1: -1, S2: -2, S3: +1
KS123-106	ttccaGAC--GTGCCGATCGCCTCgcc	S1S3: -199
	ttccaGAC--GTGCCGATCGCCTCgcc	S1S3: -199
KS123-108	ttccaGAC--TCCAGTGCTAGCTCTTcct............acaGCCCCGCATGTCTCTC--CCTtgggg............ccgGCG----GTGCCGATCGCCTCgcc	S1: -1, S2: -1, S3: -3
	-----------------CTAGCTCTTcct............acaGCCCCGCATGTCTCTC--CCTtgggg............ccgGCG----GTGCCGATCGCCTCgcc	S1: -16, S2: -1, S3: -3

Continued the table

KS123-110	ttccaGAC--TCCAGTGCTAGCTCTTcct…………acaGCCCCGCATGTCTCTCTTCCTtgggg…………ccgGCGTTCGGTGCCGATCGCCTCgcc	S1: -1, S2: +1, S3: +1
	ttccaGAC--TCCAGTGCTAGCTCTTcct…………acaGCCCCGCATGTCTCTCTTCCTtgggg…………ccgGCGTTCGGTGCCGATCGCCTCgcc	S1: -1, S2: +1, S3: +1
KS123-118	ttccaGACATTCCAGTGCTAGCTCTTcct…………acaGCCCCGCATGT-------------------------------------CGCCTCgcc	S1: +1, S2S3: -44
	ttccaGACATTCCAGTGCTAGCTCTTcct…………acaGCCCCGCATGTCTCTCT--------------------TCGGTGCCGATCGCCTCgcc	S1: +1, S2S3: -27
KS123-123	ttccaGAC-TTCCAGTGCTAGCTCTTcct…………acaGCCCCGCATGTCTCTCT-CCTtgggg…………ccgGCG-TCGGTGCCGATCGCCTCgcc	wt
	ttccaGACTTTCCAGTGCTAGCTCTTcct…………acaGCCCCGCATGTCTCTCTTCCTtgggg…………ccgGCG-----TGCCGATCGCCTCgcc	S1: +1, S2: +1, S3: -4
KS123-136	ttccaGACTTTCCAGTGCTAGCTCTTcct…………acaGCCCCGCATGTCTCTCT---------…………-------TCGGTGCCGATCGCCTCgcc	S1: +1, S2S3: -27
	ttccaGACTTTCCAGTGCTAGCTCTTcct…………acaGCCCCGCATGTCTC-----CTtgggg…………ccgGCG-----TGCCGATCGCCTCgcc	S1: +1, S2: -4, S3: -4

The mutated alleles are shown below the wild-type sequence. The distance between ERF922-S1 and ERF922-S2 is 135-bp and distance between ERF922-S2 and ERF922-S3 is 21-bp (the black apostrophes indicate nucleotides are not shown in). The target sites nucleotides are shown in black capital letters and black dashes. The red capital letters indicate inserted nucleotides and the apostrophe followed to red capital letters indicates inserted nucleotides are not shown in. The PAM site nucleotides are underlined. The red dashes indicate the deleted nucleotides. "–" and "+" indicate deletion and insertion of the indicated number of nucleotides, respectively "–/+" indicates simultaneous deletion and insertion of the indicated number of nucleotides. S1: ERF922-S1; S2: ERF922-S2; S3: ERF922-S3; S1S2: between ERF922-S1 and ERF922-S2; S2S3: between ERF922-S2 and ERF922-S3; S1S3: between ERF922-S1 and ERF922-S3; wt: wild-type sequence.
